# Characteristic of the Pepper *CaRGA2* Gene in Defense Responses against *Phytophthora capsici* Leonian

**DOI:** 10.3390/ijms14058985

**Published:** 2013-04-25

**Authors:** Ying-Li Zhang, Qing-Li Jia, Da-Wei Li, Jun-E Wang, Yan-Xu Yin, Zhen-Hui Gong

**Affiliations:** 1 College of Horticulture, Northwest A&F University, Yangling 712100, Shaanxi, China; E-Mails: zyl41982@126.com (Y.-L.Z.); jiaqingli@nwsuaf.edu.cn (Q.-L.J.); xndavid@nwsuaf.edu.cn (D.-W.L.); wjune1127@163.com (J.-E.W.); yinyanxu2008@nwsuaf.edu.cn (Y.-X.Y.); 2 State Key Laboratory of Crop Stress Biology in Arid Areas, Northwest A&F University, Yangling 712100, Shaanxi, China

**Keywords:** *Capsicum annuum* L., *Phytophthora* blight, *Phytophthora capsici* Leonian, *CaRGA2* gene, functional analysis, virus-induced gene silencing (VIGS)

## Abstract

The most significant threat to pepper production worldwide is the *Phytophthora* blight, which is caused by the oomycete pathogen, *Phytophthora capsici* Leonian. In an effort to help control this disease, we isolated and characterized a *P. capsici* resistance gene, *CaRGA2*, from a high resistant pepper (*C. annuum* CM334) and analyzed its function by the method of real-time PCR and virus-induced gene silencing (VIGS). The *CaRGA2* has a full-length cDNA of 3,018 bp with 2,874 bp open reading frame (ORF) and encodes a 957-aa protein. The protein has a predicted molecular weight of 108.6 kDa, and the isoelectric point is 8.106. Quantitative real-time PCR indicated that *CaRGA2* expression was rapidly induced by *P. capsici*. The gene expression pattern was different between the resistant and susceptible cultivars. *CaRGA2* was quickly expressed in the resistant cultivar, CM334, and reached to a peak at 24 h after inoculation with *P. capsici*, five-fold higher than that of susceptible cultivar. Our results suggest that *CaRGA2* has a distinct pattern of expression and plays a critical role in *P. capsici* stress tolerance. When the *CaRGA2* gene was silenced via VIGS, the resistance level was clearly suppressed, an observation that was supported by semi-quantitative RT-PCR and detached leave inoculation. VIGS analysis revealed their importance in the surveillance to *P. capsici* in pepper. Our results support the idea that the *CaRGA2* gene may show their response in resistance against *P. capsici*. These analyses will aid in an effort towards breeding for broad and durable resistance in economically important pepper cultivars.

## 1. Introduction

Pepper (*Capsicum annuum* L.), a member of the family, *Solanaceae*, is one of the most important vegetable crops worldwide. Phytophthora blight of pepper caused by the oomycete, *Phytophthora capsici*, is one of the most destructive diseases all over the world [1–4]. *P. capsici* attacks the roots, stems, leaves and fruits of pepper plant. It is also a pathogen of tomato, eggplant, cucumber, watermelon, pumpkin, squash and cocoa [5,6]. The pathogen can affect the plant at any stage of development causing damping-off, seedling blight, foliar blight and wilting follow by plant death. Infection of mature plants materializes as dark, rapidly expanding, water-soaked lesions [7–9].

Nearly 60 disease-resistance (R) genes have been cloned from different monocot and dicot plant species to date [10]. Plant resistance genes enable the plant to recognize the presence of specific pathogens and initiate defense responses [11]. Most of the R genes that have been characterized thus far share similar structural motifs or belong to an ancient family that encodes proteins with a nucleotide-binding site (NBS) and leucine-rich repeat (LRR) domains [12,13]. These sequences are called resistance gene homologs (RGHs) or resistance gene analogs (RGAs), due to their sequence similarity to known R genes. RGAs are abundant in plant genomes, according to sequenced genomes and PCR amplification, through degenerate primers based on conserved motifs [14]. RGA studies have been performed in the family Solanaceae [15]. The relation of RGAs to plant R genes can be described as: (1) RGAs are actual R genes [16]; (2) RGAs are linked and co-segregate with resistance gene [17,18]; (3) RGAs are not functionally related to R genes. The study of the expression of RGA genes under pathogen attack would allow for determining whether they play an active role in resistance or if they are merely linked to resistance genes [19].

Virus-induced gene silencing (VIGS) offers a rapid and high technique platform for the analysis of gene function in plants. It is a transcript suppression technique used for loss-of-function analysis of plant genes [20,21]. The technique offers an attractive alternative for knocking down target genes of interest and avoids the need for transformation [22–24]. It is considerably less time-consuming than classical stable transformation approaches. Major advances in VIGS methodology include the introduction of pTRV vectors [25,26] and the expansion of the number of VIGS hosts to include plants, such as *Capsicum annuum* [27], *Solanum* species [28], *Papaver somniferum* [29], *Aquilegia vulgaris* [30], *Eschscholzia californica* [31] and *Arabidopsis* [32,33].

*Phytophthora* blight is a devastating disease of pepper. A large number of disease resistance genes are induced by *P. capsici* invasion. Previous research focused on the conserved gene sequences of potato *RPI*(AY426259) and *Rpi-blb2*(DQ122125), using degenerate primers and rapid amplification of cDNA ends (RACE) PCR for amplifying RGAs in pepper, and then *CaRGA1*, *CaRGA2*, *CaRGA3*, *CaRGA4* and *CaRGA5* were cloned from pepper cv. CM334. Expression patterns show *CaRGA2* responses *P. capsici* differently with other RGAs in different cultivars.

In this study, we measured the effectiveness of VIGS in *Capsicum annuum* by silencing the *phytoene desaturase* (*PDS*) gene using pTRV [34]. Silencing *PDS*, which encodes an enzyme in carotenoid biosynthesis, results in white leaf tissue due to photobleaching [35]. It is a commonly used marker in VIGS experiments. Virus-induced gene silencing (VIGS) and quantitative real-time PCR of *CaRGA2* in pepper, respectively, were used to define loss- and gain-of-function of this gene in response to the oomycete pathogen *P. capsici*. Based on these results, we propose that *CaRGA2* is key factors in pathogens resistance.

## 2. Results

### 2.1. Identification of *P. capsici*

**Figure 1:**
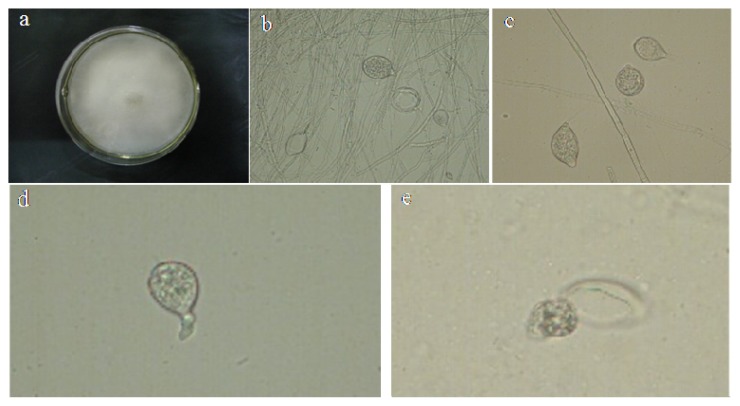
Morphology of colony and sporangia. (**a**) The colony morphology of *P. capsici* grown for seven days on potato dextrose agar (PDA) medium; (**b**) morphology of mycelial and sporangium formation; (**c**) zoosporangia (different shape and mastoid); (**d**) zoospore release; (**e**) completion of the release of zoospores.

The colony was white (Figure 1a) with dense mycelium and a regular edge after the pathogen was cultured on the potato dextrose agar (PDA) medium for three days. The mycelium was colorless, had no phragmosome and had irregular branches (Figure 1b). The variation of the sporangium morphology was greater. Most of them were orbicular-ovate, pyriform and long oval, while few were spherical or irregular, being hazel or colorless. The sporangium had an obvious mastoid (Figure 1c). Most of them had one mastoid, while few had two mastoids. The zoospores were released from the sporangium in water (Figure 1d). The pathogen resulting in the pepper Phytophthora blight in Shaanxi was identified as *Phytophthora capsici* Leonian. According to the mycelium morphology, sporangium shape, size, the disease symptom after back inoculated, the characteristic of the isolated and purified pathogen.

### 2.2. Isolation and Sequence Analysis of the *CaRGA2* Gene

The *CaRGA2* gene was cloned from a resistant pepper (*C. annuum* CM334) from leaf tissues. The *CaRGA2* cDNA (GenBank accession No. Gu116570) comprises 3,018 bp nucleotides, with a 2,874 bp open reading frame (ORF) and encoding a 957-aa protein. The initiator, ATG, is at nucleotide 22, and the stop codon, TAG, is at nucleotide 2,895. The protein had a predicted molecular weight of 108.6 kDa, and its isoelectric point was 8.106. RT-PCR products were detected by 1% agarose gel and obtained the expected size (data not shown).

### 2.3. Protein Sequence Alignment and Phylogenetics Tree Analysis

**Figure 2:**
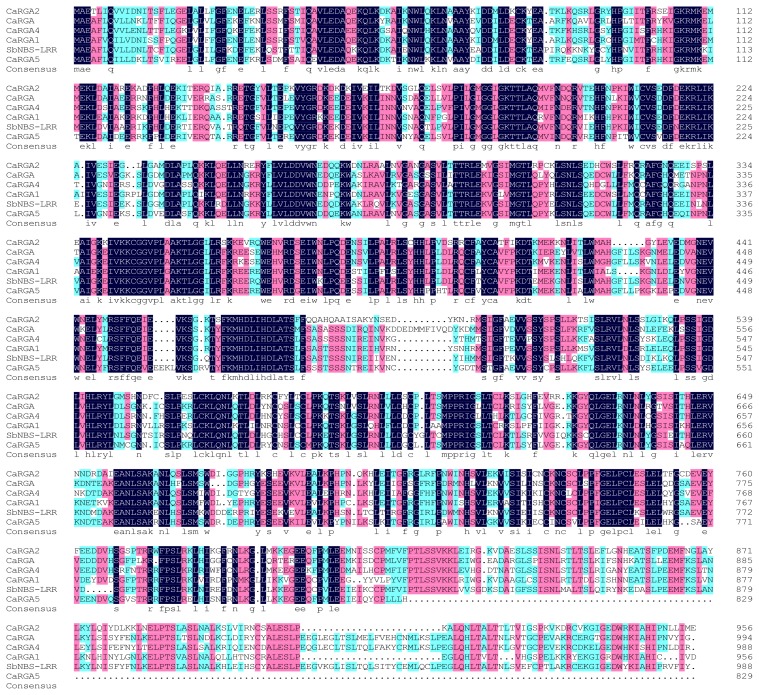
Multiple sequence alignment of the deduced amino acid sequences of *CaRGA2* gene with other resistance gene analog (RGA) resistance proteins generated with DNAMAN 6.0. Shown from top to bottom are deduced amino acid sequences of the pepper *CaRGA2* and *CaRGA* (*Capsicum annuum*, ADB43255.1), *CaRGA4* (*Capsicum annuum*, AFU51534.1), *CaRGA1* (*Capsicum annuum*, ACV53507.1), *sbNBS-LRR* (*Solanum bulbocastanum*, ACI16480.1) and *CaRGA5* (*Capsicum annuum*, AFU51535.1). Conserved residues are shaded; dots are gaps introduced to improve the alignment.

Multiple alignment of the homology of putative amino acid to five chosen sequences from GenBank database was performed using DNAMAN 6.0. Sequence alignment showed high identity with other RGA resistance proteins, including *Capsicum annuum* blight resistance protein (accession No. ADB43255.1), RGA1 (ACV53507.1), RGA4 (AFU51534.1), RGA5 (AFU51535.1) and *Solanum bulbocastanum* NBS-LRR resistance protein (accession No. ACI16480.1) at 68%, 71%, 68%, 71% and 67%, respectively (Figure 2).

**Figure 3:**
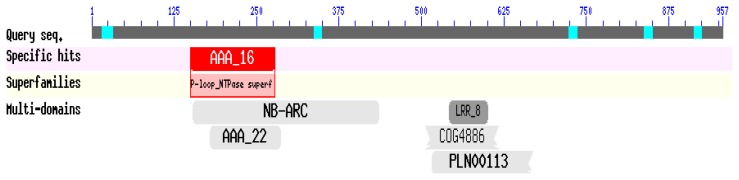
Specific conserved domains in the protein sequence of the *CaRGA2* gene.

The corresponding predicted protein (amino acid) sequence of *CaRGA2* gene was also blasted into the NCBI Blast (Protein) function for identifying homologous proteins [36]. The deduced protein belong to P-loop NTPase superfamily contains conserved domains, AAA16, NB-ARC, LRR8, AAA22, COG4886 and PLN001133 (Figure 3).

**Figure 4:**
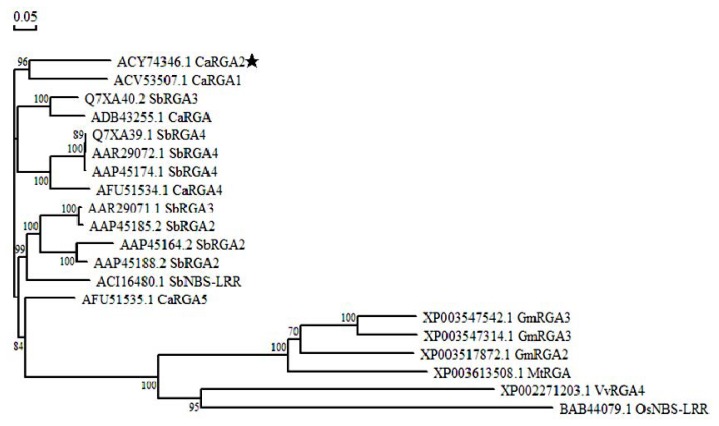
Phylogenetic relationship of *CaRGA2* to its counterparts from other plant species. Multiple alignments were performed using DNAman 6.0 program, and the phylogenic tree was created and visualized using TreeView. Protein sequences used for alignment are as follow: *Capsicum annuum* blight resistance protein (ADB43255.1), RGA1 (ACV53507.1), RGA4 (AFU51534.1), RGA5 (AFU51535.1); *Solanum bulbocastanum* Putative disease resistance protein RGA3 (Q7XA40.2), RGA4 (Q7XA39.1), RGA4 (AAP45174.1), RGA2 (AAP45185.2), RGA2 (AAP45164.2), RGA2 (AAP45188.2); *Solanum bulbocastanum* blight resistance protein (AAR29072.1), RGA3 (AAR29071.1); *Solanum bulbocastanum* NBS-LRR resistance protein (ACI16480.1); Glycine max putative disease resistance protein RGA3 (XP 003547542.1), RGA3 (XP003547314.1), RGA2 (XP003517872.1); *Medicago truncatula* resistance protein (XP003613508.1), *Vitis vinifera* putative disease resistance protein RGA4 (XP00271203.1); *Oryza sativa* NBS-LRR-type resistance protein (BAB44079.1). The tree was displayed as a phylogram in which branch lengths are proportional to distance. Numbers on the branches represent bootstrap values (for 1,000 replicates).

In order to determine the effect of the overall differences on genetic relatedness, a quantitative relationship was derived in terms of a ‘phylogenetic tree’. The phylogenetic relationship was based upon alkaline protease nucleotide sequences (Figure 4). The phylogenetic relationship among 20 RGAs from different species showed that the *Capsicum annuum*, *Solanum bulbocastanum*, *Glycine max*, *Medicago truncatula*, *Vitis vinifera* and *Oryza sativa* had a relatively close relationship. *CaRGA2* had a higher identity with *Capsicum annuum* blight resistance protein RGA1 (ACV53507.1) than other plant resistance proteins. However, the *CaRGA2* showed close relationships with all the RGAs compared to the above. This revealed that the protein structure of RGAs is highly conserved. Therefore, it is confirming the high degree of conservation of RGA during the course of evolution, which reflected the essential functions of *CaRGA2* in disease-resistance.

### 2.4. Expression Analysis of *CaRGA2* in Different Resistant Pepper Cultivars to *P. capsici*

**Figure 5:**
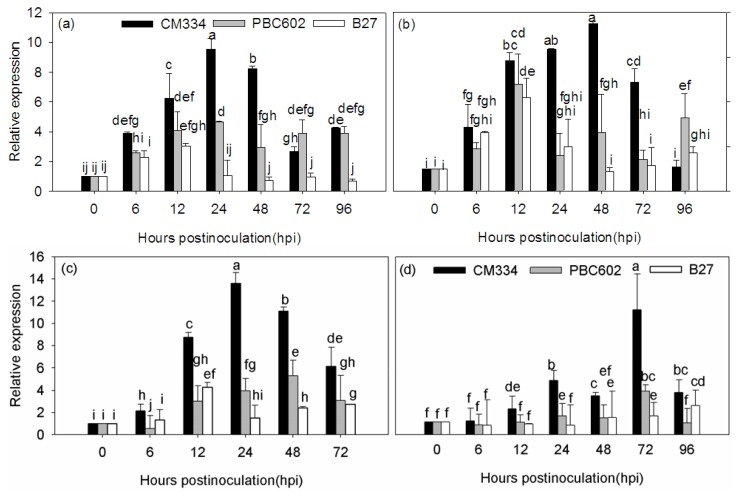
Representative quantitative reverse-transcription polymerase chain reactions (qRT-PCR) of *CaRGA2* gene. Two inoculation methods for *Phytophthora* blight resistance, one is the root inoculation method and the other is the foliar spray method. Total RNAs were isolated for analysis at different times after root inoculation with zoospores of *Phytophthora capsici* (1 × 10^5^ zoospores/mL) from leaves (**a**) and roots (**b**) in three cultivars. The same analysis used in foliar spray method for leaves (**c**) and roots (**d**) in three cultivars. qRT-PCRs were performed on cDNA using gene-specific primers for *CaRGA2* gene. Each bar represents the value of relative gene expression at different time points following inoculation of *P. capsici* for indicated defense-related gene between high resistant pepper (black bar, CM334), moderate pepper (gray bar, PBC602) and susceptible pepper (white bar, B27) plants. The expression of *CaRGA2* gene was normalized to the expression of ubiquitin-conjugating protein. Values were calculated for the *CaRGA2* gene following three replication, and standard deviations are shown.

In the root inoculation method, expression of the *CaRGA2* gene was analyzed in different tissues infected with *P. capsici* at different cultivars. The expression level of *CaRGA2* was analyzed in leaves and roots infected with *P. capsici*. The results indicated that the *CaRGA2* gene expression pattern was different between the resistant and susceptible cultivars (Figure 5a,b). The *CaRGA2* was upregulated in *P. capsici*-infected leaves and roots of all cultivars in the first 12 h followed by a decrease, while the overall expression remained lower in the susceptible cultivar (B27) when compared to controls (*CaUbi3*), whereas a higher level of gene expression was in CM334 and PBC602 cultivars. The time of gene expression was significantly affected at leaves and roots of CM334. First, the gene expression was upregulated in leaves (24 h) and roots (48 h) and then turned into being downregulated as the time increased, while the expression level in leaves was higher than roots. The highest expression level of *CaRGA2* existed in leaves and roots of PBC602 at 24 h and 12 h after inoculation, respectively. The highest expression level of *CaRGA2* existed in leaves and roots of B27 rather than other varieties, both at 12 h after inoculation. The expression level in the two tissues (leaves and roots) was basically the same. The foliar spray method showed the similar expression patterns, expressed higher in the leaves (Figure 5c) and slower in the roots (Figure 5d).

### 2.5. Enhanced Susceptibility to *P. capsici* Infection in *CaRGA2*-Silenced Plants

**Figure 6:**
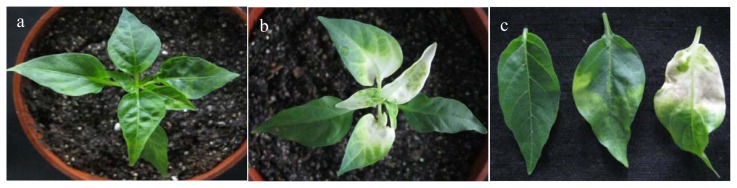
Phenotype of phytoene desaturase (PDS) silencing was observed in plant and leaves in CM334. Photograph was taken from four weeks after agroinfiltration. (**a**) was control; agrobacterium cultures transformed with tobacco rattle virus (TRV) alone were mixed in a 1:1 ratio; (**b**) was the VIGS effect of PDS on CM334 at 30 days post inoculation; (**c**) was five weeks after agroinfiltration in leaves. From left to right: non-infiltrated control, silenced (pTRV: *CaRGA2*) and silenced (pTRV: *PDS*).

We observed a strong increase in *CaRGA2* expression in response to *P. capsici* stress treatment. To further examine the effect of loss-of-function of the *CaRGA2* gene on biotic stress tolerance, we silenced the *CaRGA2* gene in pepper plants using the tobacco rattle virus (TRV)-based VIGS technique. Plants applied by pTRV empty vector were used as negative control. Tomato *PDS* genes used for initial testing and as positive controls for the silencing results. Three to four weeks after *Agrobacterium*-infiltration, *CaRGA2*-silenced pepper plants were used for experiments. Twenty days after gene silencing, pTRV-*PDS* leaves (pTRV-mediated phytoene desaturase gene silencing to yield bleaching leaves as a visualized indicator of the gene silence) exhibited a photobleached phenotype, showed adequate efficiency of the silencing system used (Figure 6), indicated viral symptoms and successful infection by the virus.

### 2.6. Semi-Quantitative RT-PCR Analysis

**Figure 7:**
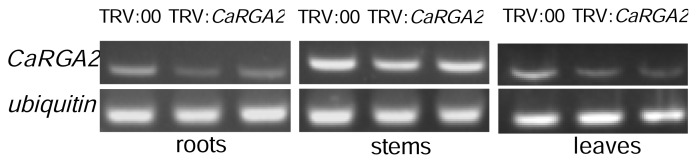
Confirmation of the *CaRGA2* gene silencing in the pTRV-*CaRGA2*-infected plants before resistance test.

VIGS efficiency was monitored by semi-quantitative RT-PCR analyses of *CaRGA2* transcript levels in an empty vector control (pTRV: 00) and *CaRGA2*- silenced (pTRV: *CaRGA2*) pepper plants (Figure 7). Transcripts of the *CaRGA2* gene in roots, stems and leaves of the recombinant pTRV-infected two independent plants at four weeks post agro-inoculation decreased differently compared to the wild-type pTRV-infected control, while the transcript level of *CaUbi3* gene used as the inner standard remained at a similar level in both types. These results confirmed silencing of the endogenous *CaRGA2* in the pTRV/*CaRGA2* plants.

### 2.7. Detached Leaves Inoculation

**Figure 8:**
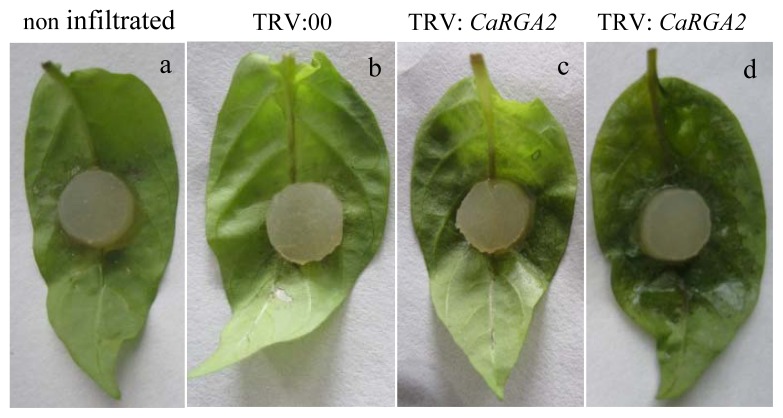
Disease symptoms developed on the non-infiltrated control, empty vector control (pTRV: 00) and silenced (pTRV: *CaRGA2*) detached leaves infected by *P. capsici*. (**a**) Non-infiltrated control; (**b**) infiltrated with TRV empty vector; (**c**), (**d**) silenced with *CaRGA2* gene (*different severity*). Twenty days after virus-induced gene silencing (VIGS) treatment, detached leaves were challenged by drop inoculation with a *P. capsici* mycelium plug and pictures were taken 72 h after the inoculation.

To assess whether the silencing of *CaRGA2* led to reduced tolerance to *P. capsici*, detaching leaves from non-infiltrated, empty vector control and *CaRGA2*-silenced plants were exposed to a 8 mm diameter mycelium plug from *P. capsici* and placing them in a petri dish containing moist filter paper followed by incubation in the dark at 25 °C for two days. A hypersensitive response (water-soaked lesions) occurred at 2 dpi on the silenced CM334 genotype upon *P. capsici* challenge, while the plants treated with empty vector produced a fairly delayed HR phenotype 3 dpi. When VIGS construct was applied to CM334 plants prior to *P. capsici* inoculation, larger and more numerous lesions with water-soaked was developed compared to the control plants (Figure 8). It was observed that non-infiltrated and empty vector control leaves inhibited disease development, showing smaller lesion size compared to the leaves of *CaRGA2*-silenced plants.

**Figure 9:**
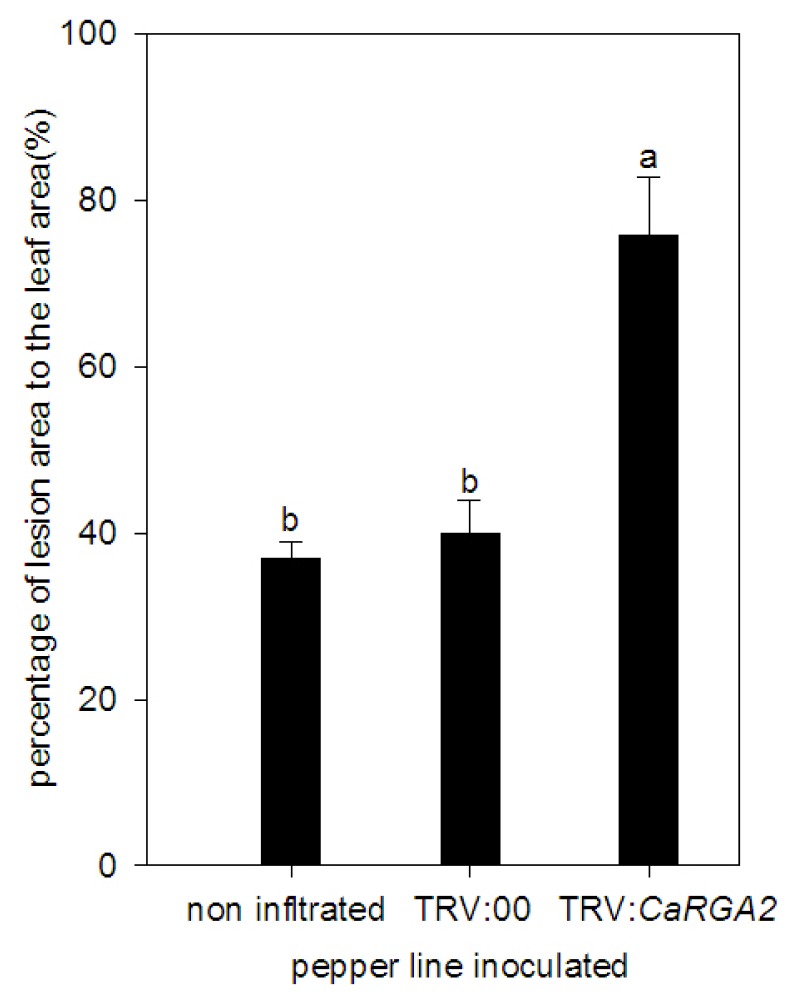
Percentage of the lesion area of the leaves inoculated with *P. capsici* isolate. Disease development was scored as the percent of foliage infected at 72 h post inoculation. Error bars represent the standard error of the mean of three independent replicates. Different letters were considered significant (*p* ≤ 0.05).

The quantitative analysis of the lesion area in the detached leaves showed a remarked increase of such an area in *CaRGA2*. The lesion sizes on detached leaves from the pTRV: *CaRGA2* inoculated plants were approximately 40% larger than those on leaves from the non-infiltrated and pTRV: 00 control plants (Figure 9). The experimental result revealed that *CaRGA2* gene knockdown resulted in increased susceptibility to *P. capsici*, and thus, *CaRGA2* is required for resistance against *P. capsici*.

## 3. Discussion

### 3.1. Identification of Pepper Phytophthora Blight Pathogen

There have been few reports on the isolation and identification of pepper *Phytophthora* blight pathogen in the regions of Shaanxi province until now. The field observations of *Phytophthora* blight of pepper in Shaanxi, China, showed that diseased plants had similar disease symptoms to that in the stage of maturity of pepper *Phytophthora* blight, *i.e.*, constricted and browning haulm, wilted leaves, sparse and white mold layer on the fruit surface. The pathogen was isolated, cultured and identified as *Phytophthora capsici* Leonia after morphological observation [37].

### 3.2. Isolation and Bioinformatics Analysis of *CaRGA2*

In this study, a *CaRGA2* gene was cloned from the pepper resistant cultivar (*C. annuum* CM334) and first isolated from the pepper. Homology analysis revealed that the *CaRGA2* gene was highly conserved in pepper and other species. Meanwhile, RGAs from pepper and other homologous species all had highly conserved LRR motifs. The phylogenetic tree and blast search showed that *CaRGA2* belongs to the LRR protein family in higher plants and had typical LRR motifs. The NB-ARC domain, a highly methionine-histidine-aspartate (MHD) motif, is present, which regulates the activity of the R protein. The knockdown gene function determined by the different domain motif away from the conserved regions. These suggested that near the 3′ or 5′-coding region is best for silencing of *CaRGA2* as a positive regulator in HR-like (hypersensitive response) cell death in pepper leaves.

### 3.3. Expression of *CaRGA2* Gene in the Different Inoculation Methods

Real-time PCR technology provides new opportunities to detect and study phytopathogenicity [38]. Because of its sensitivity, specificity and reproducibility, real-time PCR is suitable for identifying plant pathogens or for detecting minor changes in host resistance and susceptibility.

Most plant resistance genes are transcriptionally regulated in response to pathogen attack. However, due to low expression levels, transcripts can be difficult to detect using gel blot analysis [39]. The transcription of the rice *Xa1* resistance gene appears to increase following pathogen inoculation [40]. This indicates that the transcription of the R gene depends on the type of plant-pathogen interaction. In this study, the expression patterns of *CaRGA2* were investigated to gain insight into its involvement in plant defense response (Figure 5). Infection with *P. capsici* significantly enhanced the expression level of *CaRGA2*, suggesting a correlation between activating this gene and resistance to *P. capsici*. Thus, *CaRGA2* might be involved in the *P. capsici*-induced defense response. Similar expression patterns have been observed for *CSRGA23*, *Pib* and *Hs1^pro−1^*, a downy mildew resistance gene in cucumber [41], a blast resistance gene in rice [42,43] and a nematode-resistance gene in sugar beet [44], respectively.

The different inoculation methods also have an effect on the expression of *CaRGA2.* Comparison of inoculation methods for *Phytophthora* blight resistance showed highly significant correlation (*r* = 0.9150 for the root inoculation method and *r* = 0.8384, *p* < 0.01 for foliar spray method, data unpublished). Two methods can induce the expression of *CaRGA2* in different tissues.

Susceptible plants and resistant plants were inoculated with the oomycete, *P. capsici*, to evaluate the expression level of genes involved in plant defense responses over time. The highest gene expression level of *CaRGA2* in the leaves of CM334 was observed at 24 h after inoculation, while in the roots, it occurred at 48 h. The results demonstrated that the expression levels of *CaRGA2* gene were much higher in the leaves than in the roots (Figure 5). Silvar *et al*. [45] reported that the highest mRNA levels were found in the leaves, followed by the stems and roots. The tendency of gene expression was first by upward and then downward. The *CaRGA2* gene expression was different between the resistant and susceptible cultivars. The resistant cultivar CM334 showed much earlier and higher expression pattern compared to the susceptible cultivar, B27, under the pathogen stress. This expression pattern difference between the resistant and susceptible peppers might result in the difference of *P. capsici* resistance, like the *GbVe* gene expression variation in resistant and susceptible cotton resulting in the *Verticillium* wilt resistance difference [46]. Our results suggest that the difference between resistance and susceptibility depends on early detection of the pathogen. The higher level of transcript for *CaRGA2* in CM334 suggests that it is likely responsible for a large part of resistance in pepper.

### 3.4. Virus-Induced Gene Silencing of *CaRGA2* in Pepper

The virus-induced gene silencing approach has proven very useful in the analysis of gene function in plants [47–49]. VIGS can be a high throughput approach, and in contrast to conventional mutagenesis, VIGS does not alter the gene itself in the target plant, but rather transiently suppresses the expression of the gene for a specific period through degradation of specific mRNA. Conventional mutagenesis used to study the functionality of some genes can also lead to a lethal phenotype, but VIGS can be used to study the relevance of even such genes [50]. One of the specific advantages of VIGS is that the expressed sequence tags (ESTs) that are not full length can be efficiently used for silencing, and therefore, the functionality of ESTs can be studied [24,48].

To investigate the functional roles of *CaRGA2* in pepper plants during the defense response to pathogen attack, pathogen-induced *CaRGA2* gene expression was compromised in pepper leaf tissues by the VIGS technique. More recently, defense-related genes encoding pepper peroxidase, *CanPOD*, and putative integral membrane protein, *CaPIMP1*, were functionally characterized in pepper plants using VIGS [51,52]. Sarowar *et al*. [53] reported that partial suppression of the *CALTPI* or *CALTPII* gene in pepper leads to enhanced susceptibility to viral and bacterial pathogens of the pepper plant. *CaRGA2*-silenced pepper plants were slightly more susceptible to *P. capsici* compared to pTRV: 00 control plants. Lack of *CaRGA2* gene expression in silenced pepper leaves during the compatible interaction led to the induction of susceptible disease symptoms after *P. capsici* infection, accompanied by an increase in *P. capsici* proliferation in pepper leaves. These results indicate that the suppression of the *CaRGA2* gene renders the pepper plant unable to transduce a signal downstream of the broad-spectrum resistance response, thereby allowing enhanced susceptibility to pepper pathogens.

### 3.5. Semi-Quantitative RT-PCR Analysis

VIGS can knock down, rather than knock out, expression of the target gene. Our semi-quantitative RT-PCR results also demonstrated that the *CaRGA2* expression level was knocked down, rather than knocked out, in the *CaRGA2*-silenced plants, which is consistent with the nature of VIGS [50]. A high degree of silencing was observed by relative RT-PCR, which showed much lower levels of *NbACO1* transcripts compared to the controls. The result of this silencing revealed that ACC oxidase played a significant role in the interaction, as greater numbers of water-soaked lesions appeared earlier in the silenced plants than in control plants [54]. Similar results were obtained in the present research that great changes have been observed in the different tissues of leaves, stems and roots by semi-quantitative RT-PCR. The expression of *CaRGA2* shows a higher level in stems than leaves and roots. The results of semi-quantitative RT-PCR analysis also showed that the transcriptional level of *CaRGA2* mRNA significantly decreased under the conditions of VIGS infiltration, in comparison to the pTRV empty vector plants (Figure 7).

A negative correlation could be established between expression of *CaRGA2* and the lesion growth rate of the blight, *i.e.*, the higher the gene expression, the slower the disease development. This observation suggests that transcriptional regulation of *CaRGA2* may be critical to restrain *P. capsici* infection. This conclusion is also reflected by the resistance patterns of decreasing the lesion growth rate of the disease, which is a typical performance of the quantitative resistance [55,56].

### 3.6. Functional Role of *CaRGA2* in Disease Resistance

Comparative genomic studies over the past decade have established that in some species, the gene number and order are generally conserved. Moreover, many of the RGAs are species-specific or even cultivar-specific, *i.e.*, they present in only some cultivars and are absent from others within a species. There is no cultivar-specific for *C. annuum* among wild-type and cultivars (data not shown). We found that *CaRGA2* gene silencing caused enhanced susceptibility after VIGS treatment in CM334 (Figures 8 and 9). Previous studies also revealed that the *Arabidopsis atmpk4* knockout plants showed enhanced susceptibility to *A. brassicicola* [57]. Our functional analysis data demonstrate that the *CaRGA2* gene plays a role in disease resistance against *P. capsici*. This is supported by our observations that *CaRGA2* was inducible after *P. capsici* inoculation and that silencing *CaRGA2* in pepper increased susceptibility to *P. capsici*, leading to severe disease symptom and increased lesion growth. We observed that the leaves detached from the *CaRGA2* silenced plant showed an increased lesion growth rate compared to the control plants, which may be the consequence of reduced *P. capsici* resistance in the *CaRGA2* silenced plants. This suggests that enhanced expression of the *CaRGA2* gene may cause enhanced resistance to *P. capsici*.

## 4. Experimental

### 4.1. Plant Materials, Pathogen and Inoculation

*C. annuum* CM334 cultivars, *i.e.*, (high resistant), PBC602 (moderate resistance) and B27 (susceptible), were used for infection by *P. capsici*. Plants were grown in a growth chamber at 25 °C under 16 and 8 h of light and darkness, respectively. At the six leaves stage, pepper plants were inoculated with *P. capsici* [58,59].

*P. capsici* infected plants were collected from the experimental field of Northwest A & F University (Yangling, China). The pathogen was isolated from its plants by taking small cuttings from lesions at the crown. The cuttings were soaked in 75% alcohol for 30 s and rinsed with sterile water for 30 s and then 10% bleach for 2–3 min. Then, the cuttings were washed 2–3 times with sterile water and dried by sterile absorbent paper. The cuttings were transferred into the center of PDA (potato-dextrose agar) *Phytophthora-*selective plate medium (potatoes 200 g/L boil 30 min filter, agar 17 g/L, sucrose 20 g/L, penicillin 50 mg/mL, Rifampicin 100 mg/mL). From each plate showing mycelial growth, a 4 mm plug was aseptically transferred to the center of a new PDA plate medium that did not contain any antibiotics. Plates were sealed with Parafilm (Flexible Packaging, BEMIS, Neenah WI, USA) to prevent contamination and desiccation. The isolate was maintained on PDA slants at 4 °C and was grown on PDA plates at 25–26 °C in the dark.

The *Phytophthora* isolates were confirmed as *P. capsici* by observations of color and characteristics of the colony after 3 days of culturing of the purified pathogen on the PDA in dark at 25–26 °C. After that, it was incubated under continuous light for 5–7 days. The purified pathogens were overspread on the culture dish (9 cm × 9 cm) and produced a large number of sporangium. The mycelium having the sporangium was picked and placed on the slides with sterile water to observe the morphology of the sporangium under the microscope.

Zoospore release was induced by chilling cultures at 4 °C for 30 min and then incubated at room temperature for 30–60 min. Zoospore concentration was counted by using a haemocytometer and the concentration adjusted to 1 × 10^5^ zoospores/mL. The seed tray was soaked in water to keep moist before inoculating the plants. Five milliliters of spore suspension were inoculated in the 3 cm deep hole and 3 cm away from the roots of the seedlings. After inoculation, the soil was kept moist and incubated at 28 °C. Pepper plants were inoculated with the spore suspension (1 × 10^5^ zoospores/mL) using foliar spray method and then placed at a moist chamber at 28 °C. Five seedlings from each variety were used with 3 replicates. The samples of leaves and roots were taken and stored under −80 °C.

### 4.2. RNA Extraction and cDNA Synthesis

The treated leaves and roots were collected at 0, 6, 12, 24, 48, 72 and 96 h after inoculation with *P. capsici*, and total RNA was isolated using Trizol® reagent (Invitrogen, Carlsbad, CA, USA), according to the manufacturer’s instructions. Reverse transcription was carried out using a PrimeScript™ first strand complementary DNA (cDNA) Synthesis Kit (TaKaRa, Dalian, China). The concentration of total RNA and cDNA were measured spectrophotometrically using a NanoDrop instrument (Thermo Scientific NanoDrop 2000C Technologies, Wilmington, DE, USA), and the purity was assessed using the A260/280 and A260/230 ratios provided by NanoDrop.

### 4.3. Cloning of *CaRGA2*

Total RNA was extracted from leaves of four-week-old CM334 seedlings. For complete 5′ and 3′ regions of the putative *CaRGA2* gene isolation, the rapid amplification of cDNA ends (RACE) method was used. First-strand cDNA synthesis was performed using Smart RACE cDNA amplification kit (Clontech). Two 3′-RACE-gene-specific primers were designed according to the RGA fragment (GenBank accession number: Q7XBQ9.1), namely, 5′-TTCAAGAACTTTCAGTCCTCCCAATAC-3′ (3′-GSP-F1) and 5′-CAATTGTAGAATCTATTGAAGGGCTACT-3′ (3′-GSP-F2). Two 5′-RACE-gene specific primers were designed, namely, 5′-GGAGCGCAAAAGACCTCCAAGAGT-3′ (5′-GSP-R1) and 5′-ATCCAACCATTTCAAGCCGAGTAG-3′ (5′-GSP-R2). The universal primers for 5′ and 3′ RACE are given in the kit. Full-length cDNA was obtained by PCR amplification with gene-specific primers (forward primer: 5′-AGCTACCACAATATTCAACATATG-3′; reverse primer: 5′-GATACAAATCCGAAAAGATTGTT-3′).

The PCR thermal profile was set as follows: predenaturation 1 cycle at 95 °C for 5 min; 30 cycles at 95 °C for 30 s, 53 °C 3 min and 72 °C for 1 min; followed by 1 cycle of final extension at 72 °C for 10 min. The PCR products were cloned into a pMD19-T vector and sequenced.

### 4.4. Sequence Analysis and Phylogenetic Analysis

The analysis of *CaRGA2* gene sequence was done using bioinformatics tools. The nucleotide sequence, deduced amino acid sequence and open reading frame (ORF) were analyzed, and the sequence comparison was conducted through a database search using the BLAST program (NCBI, National Center for Biotechnology Services) [36]. Multiple sequence alignment was done using DNAMAN 6.0 (Lynnon Biosoft, Vaudreuil, QC, Canada) software to compare the NBS region of *CaRGA2* with other reported and characterized plant R proteins. A phylogenetic tree was constructed using neighbor-joining method with DNAMAN 6.0.

### 4.5. Primer Design and Quantitative RT-PCR for Gene Expression Analysis

Quantitative RT-PCR was performed with an iCycler iQ™ Multicolor PCR Detection System (Bio-Rad, Hercules, CA, USA). qPCR was carried out with cDNA in triplicate in 96-well plates using SYBR^®^ Premix Ex Taq™ II (TaKaRa, Dalian, China). Each reaction (20 μL) consisted of 10 μL SYBR^®^ Premix Ex Taq™ II, 2 μL diluted cDNA and 0.4 μM forward and reverse primers. qRT-PCR cycling conditions were as follows: 95 °C for 1 min and 45 cycles of 95 °C for 15 s, 52 °C for 20 s and 72 °C for 30 s. Fluorescence data were collected during the 52 °C step. As reference genes, expression of *CaUbi3* was used for pepper [60].

The accessions and primer sequences were: *CaRGA2* (GU116570; forward, 5′-TGCTAGGCGGGA AACAGGTTATG-3′; reverse, 5′-CAAGCCGAGTAGTGGTTAGAACAG-3′); *CaUbi3* (AY486137.1; forward, 5′-TGTCCATCTGCTCTCTGTTG-3′; reverse, 5′-CACCCCAAGCACAATAAGAC-3′). Relative quantification of gene expression was calculated with the Delta-Delta Ct method.

### 4.6. VIGS Plasmid Construction

The tobacco rattle virus (TRV)-derived binary vectors pTRV1 (pYL192) and pTRV2 (pYL156) have been described previously [34]. A fragment of 300 bp was amplified from the sequenced plasmid and cloned into pTRV2 vector using primers *CaRGA2*-VIGS-F (5′-GCTCTAGAATCCAACCTTAG CACTCTTACTT-3′) and *CaRGA2*-VIGS-R(5′-CGGG ATCCTCTCCTATTCCCTTCACACACC-3′), yielding pTRV2-*CaRGA2*. This construct was then introduced into the *Agrobacterium tumefaciens* strain, GV3101, by the freeze-thaw method.

### 4.7. Virus-Induced Gene Silencing (VIGS) of *CaRGA2* in Pepper

TRV (tobacco rattle virus)-based VIGS was conducted following the procedure optimized previously for the *PDS* gene silencing analysis [61,62]. We transformed with pTRV1, pTRV2-Target, pTRV2-*PDS* (*phytoene desaturase*, positive control) and pTRV2-Empty Vector (negative control) into *A. tumefaciens* strain GV3101 by the freeze-thaw method. Bacterial cells were grown at 28 °C on Luria-Bertani (LB) agar medium. The presence of pTRV1 and pTRV2 was confirmed carrying the target gene sequence by colony PCR analysis.

A single colony was selected and inoculated in 10 mL liquid culture of LB (yeast extract 10 g, peptone 10 g, NaCl 5 g/L) with appropriate antibiotics (50 mg/mL of kanamycin and 50 mg/mL of rifampicin), shaking at 200 rpm at 28 °C for 36–48 h till the OD600 reached a value between 0.8 and 1.5. Then, 2 mL of culture was inoculated into a 50 mL induction medium (IM) containing antibiotics, 10 mM MES and 200 μM acetosyringone. The culture was incubated with shaking at 200 rpm at 28 °C for 16–24 h till the OD600 reached a value between 0.5 and 1. The bacterial cells were harvested by centrifugation and resuspended individually into infiltration buffer with 10 mM MgCl_2_, 10 mM MES, 200 μM acetosyringone, pH 5.5, adjusted to an OD of 1.0 and left at room temperature for 3–5 h before infiltration into pepper plants. Each *Agrobacterium* strain containing pTRV1 and pTRV2 vectors or its derivatives (pTRV2 or pTRV2-*CaRGA2*) were mixed in a 1:1 ratio. For leaf infiltration, a mixture of the *Agrobacterium* strain containing pTRV1 and pTRV2 or its derivatives was infiltrated to the CM334 cotyledons of 2 to 3-week-old plants with 1 mL needle-less syringe. The *Agrobacterium*-infiltrated pepper plants were transferred to a growth chamber and were maintained at 17 °C for 1 d at 60% relative humidity. Then, they were placed in another growth room at 25 °C with a 16 h light and 8 h dark photoperiod cycle and used after 3–4 weeks of VIGS treatment. For the VIGS experiment, 60 lines were used for each experiment. All experiments were repeated twice.

### 4.8. Semi-Quantitative RT-PCR Analysis

Total RNA was extracted from roots, stems and leaves of wild-type CM334, silenced and non-silenced (infiltrated with empty vector pTRV1 and pTRV2) plants using Trizol^®^ reagent (Invitrogen, Carlsbad, CA, USA), according to the manufacturer’s protocols. For semi-quantitative RT-PCR, primers were designed to anneal outside the region targeted for silencing to ensure that only the endogenous gene would be detected. As reference genes, expression of *CaUbi3* was used for pepper [60].

### 4.9. Assay for Disease Resistance

A detached leaf assay was used to analyze the disease resistance of VIGS plants against the oomycete pathogen, *P. capsici*. Five detached leaves placed in Petri dishes (9 cm × 9 cm) were collected from healthy and fully-expanded leaves of 4-week-old plants infected with the non-infiltrated, empty vector control (pTRV: 00) and the silencing constructs (pTRV: *CaRGA2*). A virulent strain of *P. capsici* was grown for six days on potato dextrose agar (PDA, Difco) at 28 °C before being used for detached leaves inoculation by placing a 8 mm diameter mycelium plug from *P. capsici* onto the abaxial surface of two opposing leaves (the third and fourth true leaves). Five leaves were assayed per treatment, and the experiment was repeated three times.

Petri dishes were immediately sealed with parafilm to maintain high relative humidity and the plates incubated in an environmentally controlled growth chamber (28 °C; fluorescent light at 60 μmol·m^−2^·s^−1^ for 12-h photoperiods). Disease phenotypes and lesion sizes on the inoculated detached leaves were assessed and photographed at 72 h post inoculation (hpi).

### 4.10. Statistical Analysis

The quantitative data recorded were subjected to statistical analysis using Statistical Analysis System software (SAS Institute, version 8.2) following the one-way analysis of variance (ANOVA). Least significant difference (LSD) at *p* ≤ 0.05 was used to compare the differences among treatments. All data were expressed as the mean ± SD of three independent replicates (*n* = 3).

## 5. Conclusions

The *CaRGA2* gene was identified and its involvement in plant defense responses to *P. capsici* was characterized. Analyses of the function of *CaRGA2* by quantitative RT-PCR for gene expression analysis in CM334, *CaRGA2* gene expression was rapidly induced by *P. capsici*, suggesting a role in pathogen invasion responses. We have found out a functional role for *CaRGA2* in basal resistance and HR to *P. capsici* infection based on a reverse genetics approaches using the VIGS technique. These facts are well supported by observations that *CaRGA2* enhanced *P. capsici* disease resistance. The combination of VIGS and detached leaf assays on pepper has been proven to be a fast and powerful tool for identifying genes that may play a role in resistance to *P. capsici* in pepper. Further studies of these identified target genes functions will help us clarify the resistance mechanisms involved in the resistance to *P. capsici*. We believe that our work will help us better understand the molecular mechanisms involved in controlling *P. capsici*.
